# Hormonal Characteristics of Women Receiving Ovarian Tissue Transplantation with or without Endogenous Ovarian Activity

**DOI:** 10.3390/jcm10225217

**Published:** 2021-11-09

**Authors:** Vinnie Hornshøj Greve, Margit Dueholm, Linn Salto Mamsen, Stine Gry Kristensen, Erik Ernst, Claus-Yding Andersen

**Affiliations:** 1 Department of Obstetrics and Gynecology, Aarhus University Hospital, Palle Juul-Jensens Boulevard 82, 8200 Aarhus, Denmark; vinngrev@rm.dk (V.H.G.); margit.dueholm@clin.au.dk (M.D.); erik.ernst@dadlnet.dk (E.E.); 2 Laboratory of Reproductive Biology, The Juliane Marie Centre for Women, Children and Reproduction, University Hospital of Copenhagen, 2100 Copenhagen, Denmark; linn.salto.mamsen@regionh.dk (L.S.M.); Stine.Gry.Kristensen@regionh.dk (S.G.K.); 3 Faculty of Health and Medicine, University of Copenhagen, Rigshospitalet, Blegdamsvej 9, 2100 Copenhagen, Denmark

**Keywords:** transplantation of ovarian tissue, FSH, AMH, regaining ovarian activity, age

## Abstract

Ovarian tissue cryopreservation (OTC) and transplantation of frozen/thawed ovarian tissue (OTT) are used for fertility preservation in girls and women. Here, we evaluated the hormonal characteristics of women with or without postmenopausal levels of FSH at the time of OTT to study differences and conditions that best support the initiation of ovarian function. A total of 74 women undergoing OTT (*n* = 51 with menopausal levels of FSH; *n* = 23 with premenopausal levels) were followed by measurements of FSH, LH, AMH, and oestradiol. Concentrations of FSH and LH returned to premenopausal levels after 20 weeks on average, with a concomitant increase in oestradiol. Despite resumption of ovarian activity, AMH concentrations were in most instances below the detection limit in the menopausal group, suggesting a low ovarian reserve. Despite a higher age in the premenopausal group, they more often experienced an AMH increase than the menopausal group, suggesting that conditions in the premenopausal ovary better sustain follicle survival, perhaps due to the higher concentrations of oestradiol. Collectively, this study highlights the need for improving follicle survival after OTT. Age and the amount of tissue transplanted are important factors that influence the ability to regain ovarian activity and levels of FSH may need to be downregulated and oestradiol increased prior to OTT.

## 1. Introduction

Successful ovarian tissue cryopreservation (OTC) and fertility preservation are critically dependent on three main conditions, namely, the small size of primordial (i.e., nongrowing) follicles with a diameter of approximately 45–65 µm [1], which make them tolerable to freezing; a large number of follicles; and a favourable anatomy of the human ovary, with the majority of all primordial follicles located in the outermost one to two millimetres of the cortex [2,3]. The oocyte comprises a volume of approximately 70% of the primordial follicles with a thin layer of flattened granulosa cells covering the rest [1], making them more homogeneous and suitable for freezing. The location of the nongrowing follicles in a thin outer cortex layer makes it possible to prepare pieces of tissue that can be equilibrated relatively fast with cryoprotectants that permit the cryopreservation procedure. Furthermore, the large number of nongrowing follicles in the cortex enhances the possibility of a successful outcome. However, the conditions that facilitate freezing of ovarian tissue do not necessarily coincide with conditions that best sustain the survival of follicles in connection with ovarian tissue transplantation (OTT) [4,5]. The tissue is transplanted (often in the remaining postmenopausal ovary) without reanastomosis, leaving the tissue without a proper supply of oxygen and nutrients for the first period after grafting. Diffusion of oxygen and nutrients into the tissue requires some time to be established, during which reactive oxygen species and oxidative stress make the survival of follicles in the grafted tissue less favourable. Thus, the survival of nongrowing follicles at OTT is limited [6,7,8,9], and revascularisation of tissue in connection with OTT is an important step to improve and optimise the function of grafted tissue [10,11,12].

Revascularisation of the grafted tissue is dependent on several conditions, but the secretion of various growth factors, such as vascular endothelial growth factor (VEGF), basic fibroblast growth factor (bFGF), erythropoietin (EPO,) and others, from the follicles itself is also a critical determinant [13,14,15,16] and may also be facilitated by growth factors secreted from adult stem cells [17,18,19,20,21]. These growth factors enable the formation of new vessels and enhance access to oxygen and nutrients in a process that resembles the normal process of follicle growth initiation taking place in the ovaries in vivo. FSH receptors become expressed early in folliculogenesis, and FSH is likely to be an important hormone during OTT and in reinitiating ovarian function. However, levels of FSH at OTT are often postmenopausal and very high compared to the concentrations observed during the normal reproductive period [22,23]. Concentrations of FSH can be more than 4 to 5 times higher than those observed during ovarian stimulation with exogenous FSH. To what extent these very high concentrations of FSH are beneficial or a disadvantage during OTT is unknown. It may be that high levels of FSH stimulate growth factor production more efficiently than normal premenopausal levels or that FSH may hyper stimulate follicles to an extent that becomes a disadvantage to overall ovarian graft survival and function. The present study was designed to evaluate the hormonal characteristics of women who did or did not have postmenopausal levels of FSH prior to OTT, with the aim of clarifying some of the mechanisms that directly initiate ovarian function in a cohort of women undergoing OTT.

## 2. Materials and Methods

### 2.1. Patients

A total of 74 girls and women who had OTC and OTT at Aarhus University Hospital, Aarhus, Denmark, were included. 

The OTC was performed when women were diagnosed with a cancer disease and faced a gonadotoxic treatment—often on a short notice—and was performed irrespective of whether they were on oral contraceptives, medication, or not. Resumption of ovarian activity upon transplantation is most likely not influenced by conditions at the time of excision of the tissue, which has been shown by the fact that ovarian tissue harvested from prepubertal girls, upon transplantation in adult life, regain ovarian activity in a way similar to ovarian tissue from women who were adult at OTC [24]. The OTTs were performed by two experienced surgeons (EE and MG), and did not change over time. The thawing procedures were performed by one person (CYA), and was similar for all cases as previously published [5]. As we previously have demonstrated consistent and similar rates of follicle survival, we do not routinely perform vitality testing of the transplanted tissue [3,5].

Women with serum FSH levels exceeding 40 IU/L prior to OTT were defined as menopausal (*n* = 51, representing 54 transplantations), and women with serum levels below 40 IU/L at the time of OTT were defined as premenopausal (*n* = 23, representing 24 transplantations). The concentrations of FSH, LH, AMH, and oestradiol were followed for up to 36 weeks after OTT.

The group of patients in whom AMH was measured was reduced, as measurements of this hormone were not routinely performed at the time of OTT in the first patients of this cohort (menopausal *n* = 29; premenopausal *n* = 19).

The menopausal group included patients with the following diagnoses: breast cancer (*n* = 12), Hodgkin lymphoma (*n* = 9), non-Hodgkin lymphoma (*n* = 3), aplastic anaemia (*n* = 2), Ewing sarcoma and sarcoma (*n* = 7), cervical cancer (*n* = 3), leukaemia (*n* = 3), and various malignant diagnoses (*n* = 12). Three patients diagnosed with leukaemia had one piece of the ovarian cortex transplanted to nude mice for a period of 20 weeks to confirm that the mice did not develop cancer prior to OTT.

The premenopausal group included patients with the following diagnoses: breast cancer (*n* = 13), Hodgkin lymphoma (*n* = 2), non-Hodgkin lymphoma (*n* = 1), gestational trophoblastic disease (*n* = 3), and various malignant diagnoses (*n* = 4).

Some data have previously been published [5,24].

### 2.2. Cryopreservation and Transplantation of Ovarian Tissue

All patients had one whole ovary excised via laparoscopy in Aarhus, Denmark, and it was transported for 4–5 h on ice to the Laboratory of Reproductive Biology, Copenhagen University Hospital, Rigshospitalet, for OTC and storage. The ovarian cortex was isolated and cut into small pieces, as previously described, for slow freezing [2,3,5]. At the time of OTT, the tissue was brought (in liquid nitrogen) to the Department of Obstetrics and Gynaecology, Aarhus University Hospital, where it was thawed, and the grafting procedure was performed as previously described [24,25].

The OTC and OTT procedures were approved according to the EU tissue directive by the Danish competent authorities and by the Ministry of Health (J. no. 30–1372). All patients or parents (on behalf of their daughters) approved OTC, and all women approved OTT.

### 2.3. Follow-Up after Transplantation

All patients had uncomplicated OTTs and left the hospital on the day of surgery without having experienced any side effects. A blood sample was obtained one to two months before OTT, and patients had paused hormone replacement therapy one to two months prior to OTT. The patients were followed with blood samples and telephone consultations starting at one to three months after OTT, and approximately once per month during the following five to six months.

Hormones, including FSH, LH, oestradiol, and AMH, were measured at the Clinical Biochemistry Department at Aarhus University Hospital as normal routine samples. AMH was measured either via the Roche Elecsys assay or the DSL assay (Ansh Lab., Webster, TX, USA), with each patient having all samples measured with one assay. Thus, the results from individual patients may not be comparable to one another. However, we monitored when samples were below the assay detection limit, which was approximately the same 3 pmol/L or 0.5 ng/mL (≈3.5 pmol/L) in the different assays, as an indication of very low AMH concentrations. Furthermore, for individual patients, we monitored whether the AMH measurements increased following OTT.

For the oestradiol assay, the detection limit was 50 pmol/L, and samples below this concentration were given a concentration of 50 pmol/L for illustration purposes.

Information regarding the initiation of menstruation was obtained via telephone consultations.

### 2.4. Statistical Methods

The non- and postmenopausal group were compared using the Student’s *t*-test for normal distributed continuous variables. Categorical variables were compared with Chi-square and Fishers exact test. Regression analysis was used to compare peak oestradiol values in the two groups with age as the independent co-variable. Data were analysed using Stata software (Version 3, Stata Corp, College Station, TX, USA).

## 3. Results

### Patient Characteristics

Characteristics of the women undergoing OTT in relation to whether they had postmenopausal levels of FSH (i.e., >40 IU/L) at the time of OTT are shown in Table 1. Notably, the ages at OTC and OTT were significantly younger in the menopausal group than in the premenopausal group. In addition, the number of pieces of cortical tissue and the percentage of the retrieved tissue that were grafted were significantly increased in the premenopausal group (Table 1). The diagnosis for OTC differed between the two groups, especially in the frequency of patients with breast cancer, which was 24% in the menopausal group and 57% in the premenopausal group.

**Figure 1:**
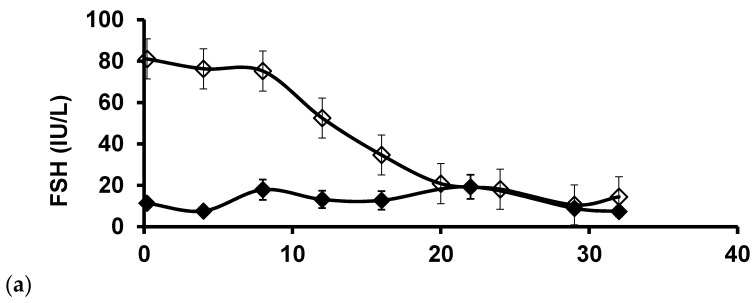

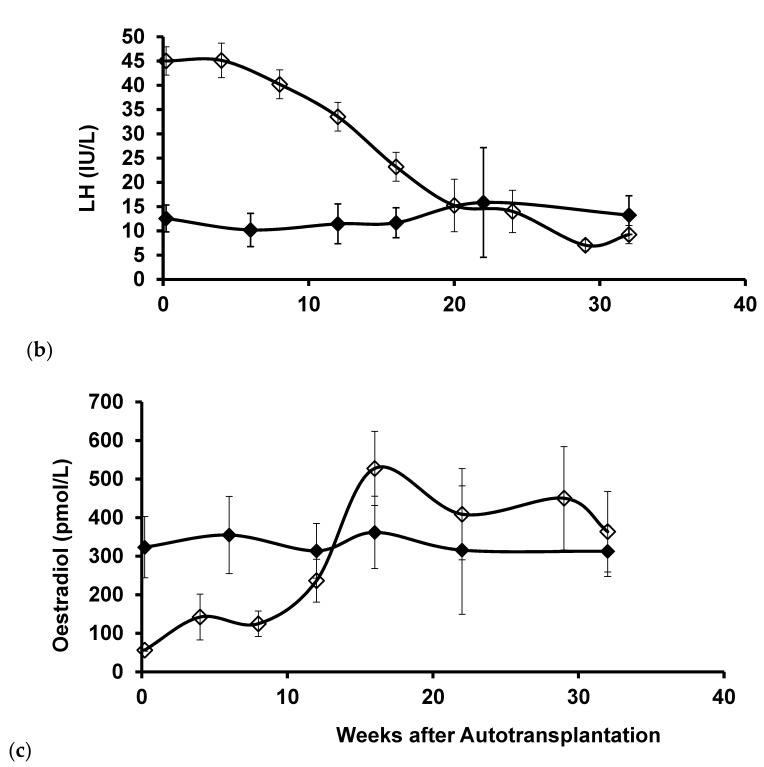
The average concentrations of FSH (**a**), LH (**b**) and oestradiol (**c**) (±SEM) during the first 40 weeks in women transplanted with ovarian tissue in relation to whether they were menopausal or not menopausal at the time of transplantation.

During the first 8 weeks after OTT, the concentrations of FSH and LH remained high in the menopausal group, where, after it gradually declined towards premenopausal levels during the following 12 weeks, it on average reached a constant level slightly above premenopausal levels (Figure 1). In the premenopausal group, the concentrations of FSH and LH were slightly elevated compared to those seen naturally and did not change significantly during the observation period.

AMH was only measured in a subgroup of patients because the assay was not available when the first cases received OTT. In the menopausal group, four out of five measurements of AMH were below the detection limit before or after OTT and could not be quantitated (Table 2), which was significantly higher than that in the premenopausal group. Notwithstanding, AMH concentrations increased in 41% of the patients following OTT (Table 2). Patients who started out with concentrations below the detection limit and subsequently showed measurable levels or women who showed an increase in the concentration measured at OTT were noted. Noticeably, patients in whom AMH concentrations increased into the measurable range were significantly younger at both OTC and OTT (*p* < 0.05) than those in which it remained undetectable, whereas the amount of tissue transplanted was similar between these two groups (Table 2).

In the premenopausal group, AMH was detected in the majority of samples, but 41% of the measurements were below the detection limit of the assay prior to OTT, resulting in two out of three patients in this group having increased AMH concentrations after OTT (Table 2).

Again, patients showing increased AMH concentrations following OTT in the premenopausal group (irrespective of whether it was detectable or not at the time of OTT) were significantly younger at both OTC and OTT than those in which it remained undetectable, whereas the amount of tissue transplanted was similar between these two groups (Table 2).

In the menopausal group, concentrations of oestradiol remained relatively low, slightly above the detection limit of the assay at approximately 10 weeks after OTT, after which, it increased to 400–500 pmol/L approximately 15 weeks after OTT (Figure 1). In contrast, the levels of oestradiol in the premenopausal group remained at a constant level between 300 and 400 pmol/L.

The concentrations of oestradiol comparing the menopausal and premenopausal groups after week 11 post OTT showed a significantly increased concentration in the menopausal group (*p* < 0.02) when age was an independent covariable (*p* < 0.02).

In the menopausal group, 41% of all oestradiol measurements were below the detection limit of the assay prior to OTT (Table 2), but all patients experienced an increase in oestradiol during the follow-up period.

All patients in the menopausal group experienced their first spontaneous menstruation at approximately 4 months after OTT as a clinical sign of endogenous ovarian function.

## 4. Discussion

This study had several noticeable findings. First, the course of FSH and LH concentrations following OTT was documented in a relatively large number of patients from one centre and confirmed that ovarian tissue on average requires approximately 20 weeks after OTT for concentrations of FSH and LH to return to premenopausal levels, extending previous observations [23,24,25,26]. Second, routine measurements of AMH in the group of patients with postmenopausal levels of FSH at OTT provide only little information on ovarian activity, since the majority of measurements are below the detection limit of the assays [11]. Third, age at the time of OTC is of utmost importance for regaining effective ovarian activity after OTT. Fourth, the pool of surviving follicles following OTT is probably low with ramifications for how much tissue should be transplanted. Lastly, the group of not-menopausal women experienced a higher chance of increasing AMH levels post-transplantation compared to the menopausal group, despite a significant higher age in the not-menopausal group. One explanation may be that the not-menopausal group experience higher levels of oestradiol during the revascularisation process, and there is good evidence that oestradiol promotes angiogenesis and growth of endothelial cells. Therefore, this suggests that providing exogenous oestradiol in the menopausal group of women may enhance revascularisation and follicles survival. Collectively, these results add to the current knowledge on how best to manage patients who receive OTT.

After OTT, concentrations of FSH and LH do not change much during the first 8 weeks, probably reflecting that only early-stage follicles survive the grafting procedure, and it takes at least two months for follicles to reach a stage where secretion of oestradiol and inhibin B reach concentrations that affect and attenuate pituitary gonadotropin secretion. Then, another 12 weeks on average elapse at which FSH and LH gradually become down regulated to premenopausal levels, evidenced by a concomitant increase in the circulatory concentrations of oestradiol and the occurrence of the first menstruation, although there is a considerable range in time period until FSH return to pre-menopausal levels. Previous morphological studies have suggested that the growth of primordial/primary follicles to the preovulatory stage normally takes approximately 5–6 months [27]. However, our current data suggest that this growth trajectory does not take quite as long as that or that a few follicles that have already embarked on growth survive the freezing procedure. The presence of healthy secondary follicles in cortical tissue after the freezing procedure has previously been documented [28]. Potentially, the very high postmenopausal concentrations of FSH after OTT may also act as a strong stimulation for follicle growth, advancing the growth rate beyond what is normally seen. Strong stimulation is probably reflected in the significantly increased concentrations of oestradiol in the menopausal group after 11 weeks of OTT compared to the not-menopausal group, showing that developing follicles in the menopausal groups are exposed to high concentrations of FSH and respond with an augmented secretion of oestradiol.

Overall, it is clear that human follicular growth is a lengthy process that requires at least four months in women transplanted with frozen/thawed ovarian tissue, although considerable variability exists in the timing of the first menstruation.

Concentrations of AMH in circulation have been proven to be a valid surrogate marker for the size of the ovarian pool of follicles [29], although the majority of AMH is produced by follicles from 4–8 millimetres in diameter [30]. In the present study, less than half of the women who were menopausal at OTT and had AMH concentrations above the detection limit of the assay experienced subsequent measurement of AMH concentrations above the detection limit, although they regained menstrual cycles. This probably reflects that only a fraction of the follicles collectively survives the OTC and OTT procedures. However, relatively few follicles are necessary for the production of one preovulatory follicle per month and thereby restore ovarian activity, especially if follicles are derived from relatively young women. Recent data show that 25% of women undergoing OTT in general become pregnant and deliver a healthy child [8], and highlight that the AMH concentration itself is a poor marker of fertility, extending previous studies [11,31]. Moreover, some of these women conceived without measurable concentrations of AMH in circulation [11]. One option to obtain more reliable AMH measurements is to use the hypersensitive AMH assay for this group of women, which has a better sensitivity than the normal employed kits, allowing detection of concentrations as low as 1.2 pg/mL (available from Ansh laboratories, Houston, TX, USA).

Calculations of the size of the ovarian stockpile of follicles that is present after OTT confirm that the number of follicles is severely reduced. Provided that one of the two ovaries are excised at OTC (in some centres, only a fraction of one ovary is preserved), then 50% of the follicular store is frozen. As suggested by the present study, approximately half of the stored tissue is currently used for transplantation to the now menopausal woman, thus representing approximately 25% of the original stockpile at OTC. The survival of follicles from the freezing procedure is approximately 80% [3], resulting in approximately 20% of the frozen supply for OTT. The ischaemia reperfusion injury that occurs in connection with OTT is probably the most ineffective step in the whole procedure, leaving, at best, only approximately one-third of the follicles surviving [6,7]. Thus, only a single digit number of surviving follicles will be available to the woman, probably approximately 5–7% of the original stockpile. Moreover, the ovaries of a normal woman 30 years of age contain approximately 5–10% of the follicles that she started out with as a newborn [32]. Thus, if the woman started out with approximately 1 million follicles at birth [32], she will, after OTT, have available in the range of 2500–7000 follicles on average, which indeed is considered to be a poor ovarian reserve and probably reflects what is observed in the present study. As a consequence, transplantation of more tissue should be considered to augment the follicle pool available for recruitment.

Collectively, this highlights that each step in the procedures should be optimised, especially the transplantation procedure, where the majority of follicles are lost for the woman. Special procedures to reduce ischaemia reperfusion injury by providing more effective strategies for follicle survival at OTT should be a focus.

It is well known that the stockpile of follicles is heavily influenced by age, and the present study clearly demonstrates that advanced age is associated with the risk of seeing no increase in AMH after OTT. This again highlights that age at OTC is a major determinant of how effectful OTT is, and that age at OTC should be considered in the context of planning OTT. Considering the above calculations on the remaining number of follicles following OTT, we have now started to transplant more tissue (and sometimes all the stored tissue) to women above the age of 30 years at OTC. The effect of this changed strategy for OTT is likely to also be observed in the not-menopausal group of patients. Here, the amount of tissue used for OTT was increased from 44 to 56% of one ovary compared to the menopausal group. In the not-menopausal group, the frequency of patients who experienced an increased AMH concentration increased from 41 to 68%, despite a significantly increased age. This may reflect the increased number of follicles being transplanted or that an ovary that is still active provides a better environment for transplantation of ovarian tissue. Additionally, premenopausal concentrations of FSH may cause less stressful stimulation of the follicles than postmenopausal concentrations in the menopausal group and provide better survival. The increased concentrations of oestradiol immediately from the time of OTT may also facilitate more effectful neovascularisation. Whereas oestradiol is known to have no effect on granulosa cell proliferation and the development of follicles in women [33], oestradiol is known to induce angiogenesis in several target organs by stimulating positive regulators of angiogenesis, and oestradiol is known to promote vasodilation in the peripheral vasculature and in coronary arteries [34,35,36,37]. Oestradiol decreases the response of blood vessels to damage [34]. It can be hypothesised that higher oestradiol concentrations in circulation and in the ovary attenuate oxidative stress and apoptosis and augment the growth of new vessels in the stroma, thereby facilitating reimplantation. Collectively, more careful studies are needed to examine the role of oestradiol during the revascularisation of grafted cortical tissue.

Furthermore, it is noticeable that three out of four patients in the not-menopausal group were former breast cancer patients, who often were older than other patient groups at OTC and OTT. The present study is unable to provide information on whether this diagnosis is associated with the higher frequency of patients who see an increase in AMH concentrations. Our data show that breast cancer patients only become menopausal to a limited extent due to the treatments related to the diagnosis, but they return for OTT to increase their ovarian reserve and chances of becoming pregnant. More patients are needed to determine whether OTT will improve their fertility.

We used 40 IU/L FSH to separate the menopausal women from the not-menopausal. A cut-off of 25 IU/L FSH has been suggested in for instance the ESHRE guidelines and could have been used. However, this is a special group of women being exposed to gonadotoxic treatment. In the group of not-menopausal women, the ovarian reserve is, after treatment, often severely compromised, although the ovary may still be active. These women fluctuate in their FSH levels and not infrequent concentrations may exceed 25 IU/L to return to the normal pre-menopausal levels when the next follicle reaches the oestrogen secreting stage. However, FSH levels seldom exceeds 40 IU/L without being post-menopausal, which is the reasoning behind choosing 40 IU/L as a cut-off in the current study.

One limitation of this study was that it was not appropriate to follow the reproductive outcomes after OTT in women in the premenopausal group because it is impossible to know whether the oocyte that results in a pregnancy derives from the transplanted tissue or from a follicle located in the remaining endogenous ovary. Furthermore, the number of women conceiving in the menopausal group is too small for meaningful comparisons, especially considering that women may become pregnant even 5 years after OTT and, therefore, change from the nonpregnant to the pregnant group [38].

Although this is probably the largest study until now, it is a limitation that the overall number of patients being included in this analysis is limited, and more data on more patients are required to make substantial conclusions.

Two different assays were used to monitor AMH concentrations in this study. The two assays are not comparable one to one, but the lower limit of detection is almost similar (around 3 pmol/L), and the measure used in this study is the number of measurements that are below the detection limit. Therefore, the impact of using two assays is not likely to affect the results and conclusions drawn from these measurements.

The current study does not address the functional longevity of the grafts; indeed, transplantations to this group of patients still require long-term follow-ups to monitor whether age is able to predict the lifespan of ovarian activity in connection with OTT.

In conclusion, the current data in combination with previously published data support that it takes approximately 20 weeks for FSH and LH to reach premenopausal levels on average. The number of follicles transplanted during OTT is probably lower than anticipated, and the transplanted tissue is often unable to cause an increase in the concentration of AMH in circulation. Thus, these results suggest that a larger pool of follicles should be transplanted, especially women in their thirties, and the age of women at OTC should preferentially be as low as possible.

Interestingly, significantly more women experienced an increase in AMH concentrations in the premenopausal group than in the menopausal group, which suggests that reducing levels of FSH and increasing levels of oestradiol prior to OTT may be beneficial for follicle survival and maintaining a larger ovarian reserve, but more studies are needed to confirm this hypothesis.

## Figures and Tables

**Table 1:** Characteristics of women transplanted with ovarian tissue in relation to whether or not they had postmenopausal levels of FSH (>40 IU/L) at the time of transplantation.

	No. Women	Age at OTC (years)	No. Cortical Pieces Frozen	Ovarian Volume (mL)	Age at OTT (years)	No. Pieces Transplanted	% of One Ovary Grafted *
Postmenopausal (FSH: >40 IU/L)	51	26 ± 1.1 * (9–39) (27.6)	23 ± 1.1 (9–49) (22)	6.6 ± 0.5 (1.8–12.2) (6.0)	31 ± 0.8 * (14–42) (31.2)	9.5 ± 0.4 * (2–16) (10)	43 ± 1.7 * (18–77) (42)
Not menopausal FSH: ≤40 IU/L)	23	30 ± 1.2 * (15–37) (30.7)	21 ± 1.2 (8–31) (21)	7.0 ± 0.8 (2.1–18.3) (6.2)	35 ± 1.1 * (26–44) (35.9)	11 ± 0.9 * (5–22) (10)	59 ± 4.3 * (21–100) (55)

Values are: Mean ± SEM (range) (median); * Students *t*-test: *p* < 0.05; OTC: Ovarian tissue cryopreservation; OTT: ovarian tissue transplantation; FSH: Follicle Stimulating Hormone.

**Table 2:** Serum AMH concentrations of women transplanted with ovarian tissue in relation to menopausal status at the time of transplantation, age and amount of tissue grafted.

		No. Women	AMH Measurements below DL Prior to OTT	Pt. with AMH Increase	Age at OTC	Age at OTT	% Tissue Grafted		Age at OTC	Age at OTT	% Tissue Grafted
AMH	Postmenopausal	29	81% *	12 (41%)	26 ± 1.7 (9–38)	31 ± 1.2 (20–42)	44 ± 2.5 (20–77)	AMH increased	22 ± 2.7 ** (9–22)	28 ± 1.8 ** (20–37)	49 ± 2.7 (29–64)
								No increase	29 ± 2.0 ** (9–39)	36 ± 1.6 ** (20–37)	43 ± 3.9 (20–77)
	Non-menopausal	19	41% *	13 (68%)	30 ± 1.4 (15–37)	35 ± 1.3 (26–44)	56 ± 4.5 (24–100)	AMH increased	27 ± 1.9 ** (15–37)	33 ± 1.6 ** (26-41)	55 ± 5.4 (24-100)
								No increase	33 ± 1.4 ** (26–37)	39 ± 1.6 ** (31–44)	59 ± 8.3 (24–96)

DL: Detection Limit; * Chi-square test: *p* < 0.05; ** Students *t*-test: *p* < 0.05; AMH: Age in years.
